# Bilateral Deep Vein Thrombosis (DVT) as a Harbinger of Lung Adenocarcinoma: A Rare Presentation

**DOI:** 10.7759/cureus.44229

**Published:** 2023-08-27

**Authors:** Saleh A Ba-shammakh, Obada A Al Jayyousi, Mohammad Abu-Hussein, Mahmoud M Abokhsab, Mohammad H Al-thnaibat, Hasn M Haj-Freej, Abdulrahman M Al-Bourah

**Affiliations:** 1 General Surgery, The Islamic Hospital, Amman, JOR; 2 Medicine, Jordan University of Science and Technology, Al Ramtha, JOR; 3 General Surgery, Jordan University of Science and Technology, Al Ramtha, JOR; 4 Internal Medicine, The Islamic Hospital, Amman, JOR

**Keywords:** carcinoembryonic antigen (cea), thrombophilia screen, entrectinib, vte, coagulation cascade, bilateral dvt, lung adenocarcinoma, deep vein thrombosis (dvt)

## Abstract

Oncologic disorders, such as lung adenocarcinoma, can intricately interplay with the coagulation cascade, often leading to thromboembolic events, of which deep vein thrombosis (DVT) stands out prominently. In this report, we present a unique case of a 50-year-old non-smoking Jordanian male who exhibited bilateral DVT as an unexpected preliminary manifestation of an aggressive lung adenocarcinoma. Although the patient did not possess common risk factors for DVT, the bilateral presentation drew attention to the possibility of an underlying malignancy. Subsequent investigations revealed a stage 4 primary lung adenocarcinoma. This case underscores the imperative of maintaining a broad differential in cases of DVT, especially when presenting bilaterally and without evident etiology. Such early detection and intervention, accompanied by collaborative medical strategies and specialized care, can play a pivotal role in enhancing patient prognosis and survival rates. This case exemplifies the potential of DVT, particularly when bilateral, as a harbinger of a more sinister underlying pathology like lung adenocarcinoma.

## Introduction

Oncologic disorders often interact intricately with abnormalities in the coagulation cascade, leading to various thromboembolic events, including deep vein thrombosis (DVT), pulmonary embolism (PE), arterial thrombosis, nonbacterial thrombotic endocarditis, and ischemic strokes. These events, while not typically the primary indicators of an unseen malignancy, often surface more prominently as the malignancy advances. They can serve as early alert signals due to the intricate link between malignancies and coagulation cascade disturbances [1].

Deep vein thrombosis stands out as a commonly encountered venous thromboembolic (VTE) condition, with an annual incidence of 80 cases per 100,000 and a prevalence rate of lower limb DVT at 1 per 1,000 individuals [1]. Recognized risk factors for DVT encompass obesity, pregnancy, age above 60 years, surgical procedures, admissions to critical care units in hospitals, dehydration, and notably, cancer [2-3]. Lung cancer had the highest incidence of venous thrombosis after brain cancer [3].

The extent of site involvement is primarily influenced by the anatomical position, with distal veins at 40%, popliteal at 16%, femoral and common femoral both at 20%, and iliac veins at 4% [4]. Fürbringer-Schwarz et al.'s 2022 study revealed that 19.1% of thrombosis cases had bilateral DVT and were more common than unilateral DVT in cancer [5]. While this percentage is slightly higher, it remains consistent with earlier findings by Markel et al. in 1992, where they recorded a rate of 17% for bilateral DVT [6]. It is also reported that thromboembolism in the context of cancer constitutes around 20% of thromboembolic occurrences with lung cancer posing a higher risk. Notably, post-bilateral DVT mortality was most frequently attributed to cancer at 38%, which was only slightly ahead of malignant tumors at 26% after unilateral DVT [5]. This case study delves into an instance where a bilateral DVT was the preliminary manifestation of hidden aggressive lung cancer in a middle-aged male.

## Case presentation

A 50-year-old Jordanian male, non-smoker, with a known history of hypertension for four years, presented to our Emergency Department with chief complaints of bilateral leg pain persisting for the past week. The pain was predominantly in the right calf, exhibiting a gradual onset and progressive nature. It was described as sharp, with a severity of 7/10 using a visual analog scale (VAS), and localized mainly around the right ankle joint. Furthermore, the pain was aggravated by movement and relieved upon intake of celecoxib. There was no associated history of weight loss, night sweats, fatigue, or febrile sensations. The patient also denied having oral or genital ulcers, skin rashes, or symptoms suggestive of morning stiffness. He did, however, mention a positive family history of scleroderma.

Upon admission, the patient was calm and cooperative. His vitals were as follows: blood pressure 122/80 mmHg, heart rate 76 beats/min, respiratory rate 16 breaths/min, body temperature 36.8°C, and oxygen saturation at a stable 98% on room air. A systemic review was largely unremarkable, except for a notable right ankle joint swelling and pain. Despite the swelling, the ankle joint had a full range of motion without evident redness, heat, or surrounding skin abnormalities.

Preliminary laboratory investigations returned a normal complete blood count, kidney, and liver function. However, there was a noted elevation in inflammatory markers. The rheumatological panel was normal, despite the patient's family history of scleroderma. Coagulation profile results were as follows: prothrombin time (PT): 15.2 s, partial thromboplastin time (PTT): 28 s, and international normalized ratio (INR): 1.16 s. The uric acid level was within normal parameters.

**Figure 1:**
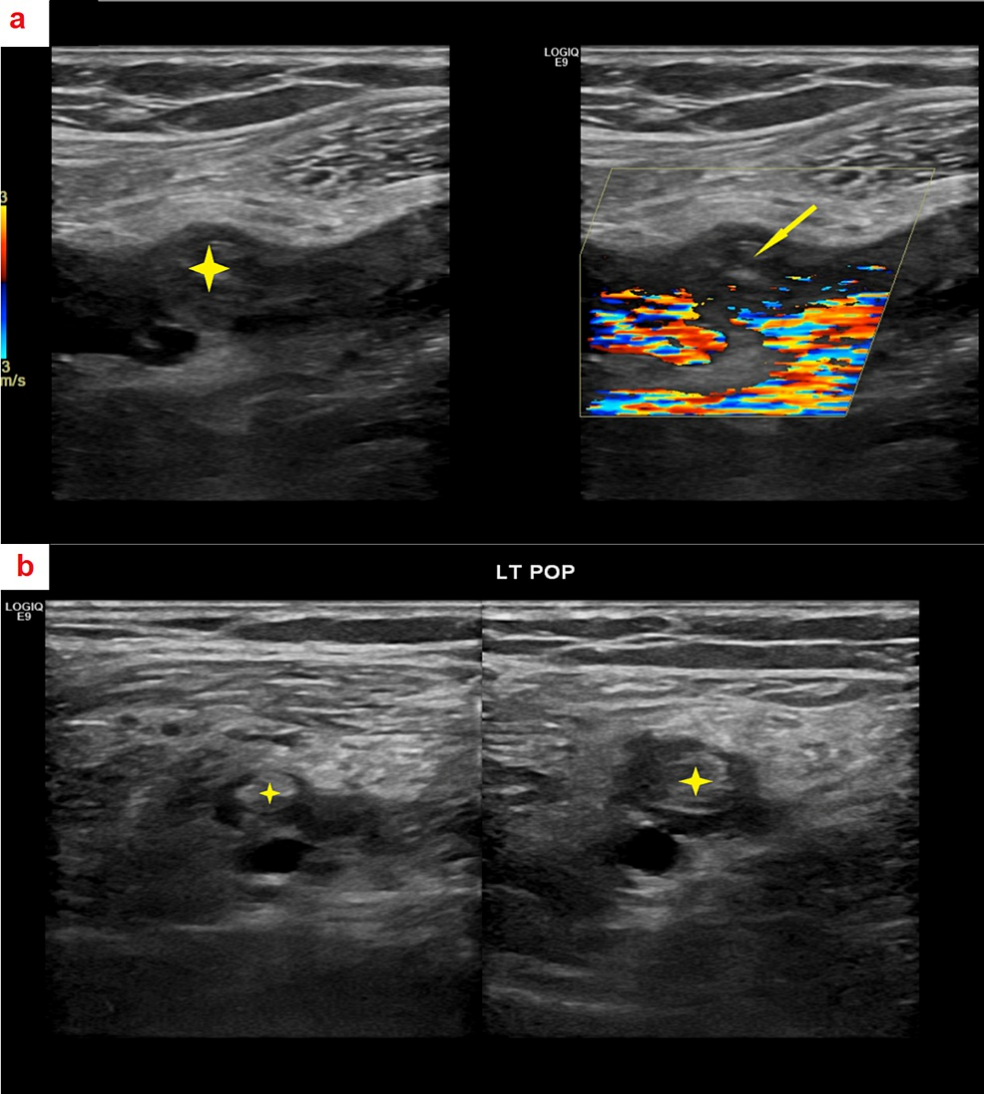
Bilateral lower limb Doppler imaging. a-b: Acute DVT in the left distal superficial femoral, popliteal, and portions of the left and right posterior tibial veins. Superficial thrombophlebitis in a segment of the right greater saphenous vein below the knee. Notable subcutaneous and interstitial edema in the right lower leg. DVT, deep vein thrombosis

A bilateral lower limb Doppler (Figure 1a,b) revealed acute DVT in the left distal superficial femoral, popliteal, and visualized parts of the left posterior tibial and intramuscular branches of the right posterior tibial veins. In addition, superficial thrombophlebitis was found in a long segment of the right greater saphenous vein below the knee. Subcutaneous and interstitial edema was also observed in the right lower leg.

**Figure 2:**
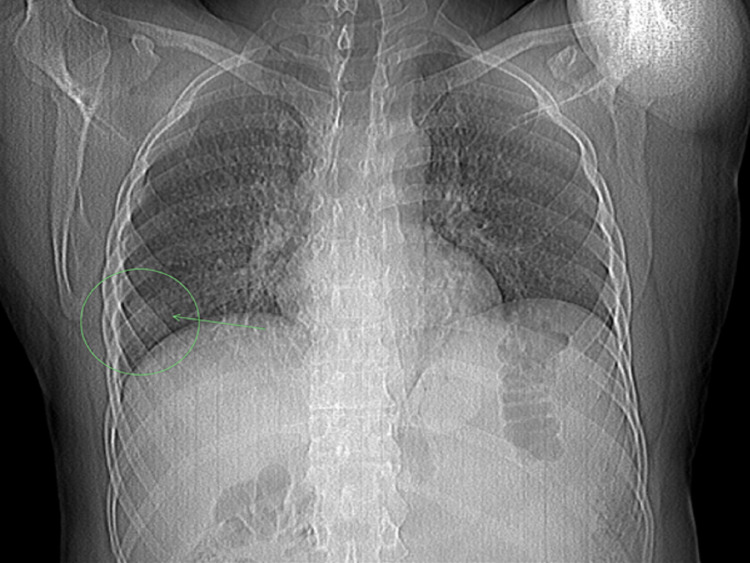
Chest X-ray. Demonstrates a well-defined nodule in the right lower lobe accompanied by a surrounding ground-glass opacity.

**Figure 3:**
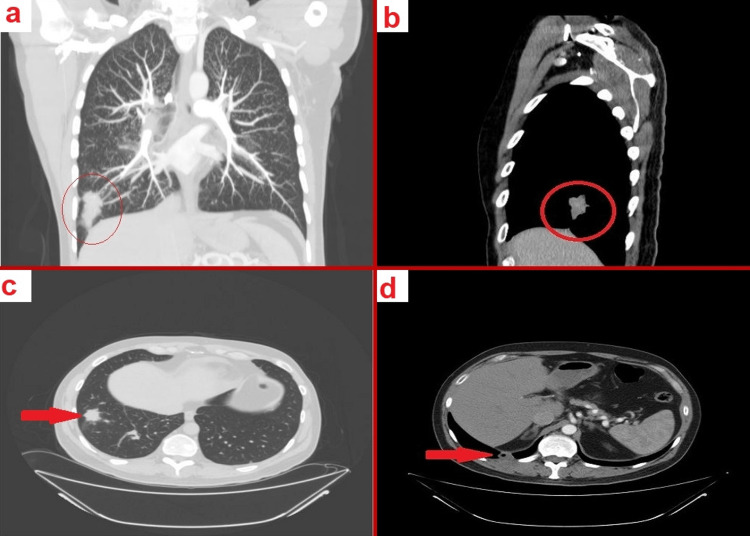
CT scan of the chest, abdomen, and pelvis with IV contrast. a-d: Revealing lung lesions, and tiny centrilobular lung nodules, suggesting possible miliary metastasis.

Following this, a chest X-ray was undertaken (Figure 2), prompting the patient's immediate referral to the hemato-oncology clinic. CT scans of the chest, abdomen, and pelvis with IV contrast displayed two distinct hypoattenuating nodules in the left thyroid lobe, a spiculated mass lesion, and a cavitary lesion in different segments of the right lower lung lobe. Also noteworthy was the presence of bilateral miliary distributed tiny centrilobular lung nodules, hinting at possible miliary metastasis, and a minimal pericardial effusion. There were no findings suggestive of pulmonary embolism (Figure 3a,d).

**Figure 4:**
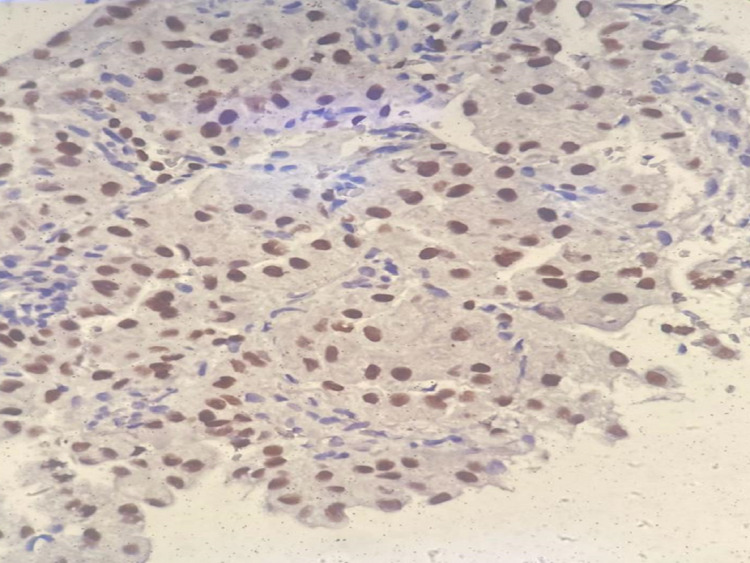
Immunohistochemical staining for TTF-1. Microscopic view of lung biopsy specimen stained positive for TTF1 with atypical large cells with irregular nuclei and prominent nucleoli are prominent. 40x magnification TTF-1, thyroid transcription factor 1

Based on the imaging results, a CT-guided lung biopsy was promptly performed. Histological examination of the biopsy specimen revealed lung parenchymal tissue infiltrated by atypical large cells with irregular nuclei and prominent nucleoli. These cells had tendencies to form glandular structures and showed occasional mitotic figures, aligning with a diagnosis of adenocarcinoma. Immunohistochemical staining (Figure 4), which showed positivity for TTF1 and Napsin A but negativity for CDX2, confirmed the diagnosis as a primary lung adenocarcinoma of stage 4.

Given the diagnosis and the patient's overall clinical status, he was initiated on a treatment regimen that included Entrectinib as decided upon a multi-disciplinary team meeting. Entrectinib was chosen as the biopsy was ROS1 positive. The patient responded positively to the initial rounds of treatment, with a marked reduction in leg pain and swelling of the ankle joint. Subcutaneous injections of low molecular weight heparin, 80 mg twice daily, were the mainstay of treatment for DVT. Regular follow-ups were scheduled to monitor the effectiveness of the therapy and any associated side effects.

However, given the extent and nature of the disease and its inherent complications, the decision was made to transfer the patient for further specialized care. Considering the case's complexity and the need for a multidisciplinary approach, the patient was transferred to the King Hussein Cancer Center (KHCC) to continue his treatment. This esteemed facility specializes in oncology cases and has a comprehensive care approach equipped to manage advanced-stage adenocarcinoma of the lung.

At KHCC, the patient was seen by a team of specialists, including oncologists, pulmonologists, radiologists, and surgeons. He underwent further diagnostic tests, including molecular profiling, to evaluate the potential efficacy of targeted therapy, especially considering his current treatment regime with Entrectinib.

His family was counseled about the aggressive nature of the disease and the potential outcomes. The importance of a robust support system and palliative measures, if required, were also discussed in detail. The patient remained under the care of KHCC, with regular visits to assess disease progression, treatment efficacy, and overall quality of life.

While the journey was challenging, the combined expertise from both healthcare facilities ensured that the patient received the best possible care, exemplifying the significance of specialized centers and collaborative medical efforts.

## Discussion

Lung adenocarcinoma is typically present in the periphery and, therefore, is usually asymptomatic at early stages and diagnosed incidentally on imaging. In our case, the patient presented with bilateral DVT, which is rather unusual, especially with a negative thrombophilia screen [7].

There is some evidence in the literature favoring an increased risk of DVT in general with lung adenocarcinoma. However as opposed to our case, a study reports a higher association with younger age [8].

A few case reports also report a similar presentation, where one study describes the condition of a patient with lung adenocarcinoma, presenting with various thromboembolic events [9]. In other studies, cerebral vein thrombosis and numerous infarcts were the presenting features of lung adenocarcinoma [10-11]. Interestingly too, a diagnosis of metastatic lung adenocarcinoma has been established in a patient with recurring thromboembolic occurrences despite anticoagulation [12]. All of which support an association between lung adenocarcinoma and thromboembolism, however, none of which noted bilateral DVT. Carcinoembryonic antigen (CEA) is believed to play a key role in the link between lung adenocarcinoma and thromboembolism [8].

Venous thromboembolism (VTE) in a picture of cancer is unfortunately associated with a worse prognosis in terms of mortality and long-term survival [13]. So, appropriate treatment and prophylaxis are necessary. Regarding cancer-related VTE, LMWH has been reported to be superior to warfarin and direct oral anticoagulants (DOACS) for treatment [14]. VTE prophylaxis with LMWH has also been encouraged pertaining to a lower VTE risk with the absence of higher chances of bleeding or death [15].

Upon suspicion of lung adenocarcinoma and for patients with multiple risk factors, screening is advised via low-dose CT according to the US Preventative Services Task Force [16]. Positron emission tomography/CT (PET/CT) scan and a biopsy are recommended afterwards if there were to be any suspicious lung nodules. In our case, a suspicious lesion was apparent on chest X-ray, and thus was followed by a CT scan.

CDX2, Naspin A, and TTF1 are well-reported markers in the workup of adenocarcinoma and all of which have been investigated for in our patient. It is suggested that patients with higher TTF1 and Naspin A expression might have a better prognosis [17-18].

Our case report highlights the importance of considering malignancy among the differential diagnosis of bilateral DVT and lung adenocarcinoma particularly if the clinical picture applies. This is crucial to help prevent any further diagnostic delay with the aim of improving mortality and long-term survival.

## Conclusions

This case underscores the potential of bilateral DVT as an early manifestation of aggressive lung adenocarcinoma, particularly in patients without common risk factors for thrombosis. While DVT is a recognized complication of malignancy, bilateral presentation as a preliminary indicator is relatively rare. Our findings emphasize the importance of keeping a broad differential in cases of DVT and recommend further investigations when there is an unexplained clinical presentation. Given the association between malignancy and thrombosis, early diagnosis and intervention are crucial to improve prognosis and overall survival rates. Collaborative medical efforts and specialized care can greatly enhance patient outcomes, as demonstrated in this case.
